# Gamifying Parenting Education Using an App Developed for Pacific and Other New Zealand Families (Play Kindly): Qualitative Study

**DOI:** 10.2196/15647

**Published:** 2020-06-10

**Authors:** Rebecca A Mairs, Marthinus J Bekker, Tony Patolo, Sarah A Hopkins, Esther T Cowley-Malcolm, Lana M Perese, Gerhard B Sundborn, Sally N Merry

**Affiliations:** 1 Department of Psychological Medicine University of Auckland Auckland New Zealand; 2 Malatest International Auckland New Zealand; 3 Department of Pacific Health University of Auckland Auckland New Zealand

**Keywords:** parenting, mHealth, Pacific peoples

## Abstract

**Background:**

Play Kindly is a gamified animated app designed to address common behavioral problems in childhood. The interface is designed to appeal to Pacific people, a population group with a higher risk of developing clinically significant behavioral problems than most other ethnic groups in New Zealand.

**Objective:**

The aim of this study is to explore the opinions of parents and professionals about the acceptability, usability, and content of Play Kindly.

**Methods:**

We used qualitative and Pacific and Māori research methodologies. A total of five focus groups with 45 parents and 12 individual interviews with professionals were conducted. The five focus groups consisted of 2 pan-Pacific groups, 1 Māori group, 1 open group, and 1 group of young Pacific adults or prospective parents. The professionals were from a range of disciplines, and the majority had expertise in early childhood, parenting interventions, or research in this field.

**Results:**

Play Kindly appealed to both parents and professionals. Participants related to the scenarios, which were created in collaboration with a playwright and animator. Although most participants liked the Pacific feel, there was some disagreement about how culturally specific the app should be. A range of issues with usability and gamification techniques were highlighted, likely attributed to the low budget and lack of initial co-design with parents as well as professionals with specific expertise in parenting. A number of parents and professionals felt that the parenting strategies were overly simplified and did not take into account the context in which the behavior occurred. Professionals suggested narrowing the focus of the app to deliver two important parenting messages: playing with your child and positively reinforcing desired behaviors.

**Conclusions:**

Play Kindly is the first culturally adapted parenting app of its kind designed for Pacific parents and other New Zealanders with children 2-5 years of age. This app has potential in Pacific communities where there are limited culturally specific parenting resources. The results of this study will guide improvements of the app prior to testing it in an open trial.

## Introduction

O fanau a manu e fafaga i fuga o laau, a o tama a tagata e fafaga i upu.

“The young ones of birds are fed with nectar; the children of people are fed with words” [1].

Behavioral problems are common at some time in a child’s development. The way parents respond to these play a major role in how likely the child is to repeat the behavior. If behaviors start to fall outside the normal range for the child’s age and stage of development, they are referred to as disruptive behavior disorders. These disorders can cause difficulties with peer relationships, school performance, aggression, and eventually substance abuse and criminality [2,3].

A number of evidence-based interventions have been shown to be effective for the prevention and treatment of disruptive behavior disorders. Early intervention with parent management training has been one of the most successful approaches in early and middle childhood [4]. In this training, facilitators teach parents a range of skills for the management of behavior. Unfortunately, there are often barriers for accessing these programs. For example, participating in parenting programs can mean loss of time spent with children and loss of earnings, which is particularly important for families facing economic hardship [5]. In New Zealand, like many other parts of the world, availability of these programs is limited by underfunding and a lack of expertise in the workforce for their delivery. There are a number of rural communities where access to consistent health care is challenging [6].

Online parenting programs have shown some positive effects on parenting strategies and skills [7-9] relative to control groups and compared with face-to-face parent seminars and bibliotherapy approaches; however, this evidence is modest. Online, gamified parenting interventions delivered on mobile phones and targeted to vulnerable communities have demonstrated reduced behavioral difficulties in children, an increase in more effective parenting strategies, and decreased parental stress [10]. Digital programs could, therefore, address problems with workforce shortages, availability of parents, and geographical isolation. In fact, consumer preference research shows that vulnerable families prefer online resources as a way of receiving parenting support [11,12].

In New Zealand, approximately 20% of children have a medium to high likelihood of having disruptive behavior [13]. Although this figure is comparable to international findings [4], the risk is higher for Pacific (24%) and Māori (28%) children [13]. One of the reasons is that a higher proportion of these children live in poverty, and the challenges of economic hardship can lead to difficulties in parenting effectively [6]. There are few parenting programs that cater specifically to Pacific and Māori parents. Despite the fact that these groups are considered to be more at risk and that retention is better in parenting programs that are specific to a culture [14], there are few programs with a focus on targeting these populations.

Pacific groups are the fourth largest major ethnic group in New Zealand [15], and the number of Pacific children is projected to increase at a higher rate than any other ethnic group in New Zealand [15]. A recent study [16] found that compared to Samoan parents in Samoa, Samoan parents living in New Zealand were more authoritarian, especially fathers, who were more likely to undertake harsh discipline. This may be due to the lifestyle in New Zealand where alcohol, drugs, and other risk factors are more prevalent [16]. New Zealand born Samoan and Pacific parents who were more highly educated and had good levels of support were more likely to discard the harsh discipline practices of their parents [17,18].

“Play Kindly” is the first app in New Zealand designed for a contemporary Pacific audience that uses gamification to teach parenting strategies for everyday behavior problems. The aim of this study was to carry out a qualitative investigation using Pacific and Māori research methods to assess the acceptability, feasibility, and perceived utility of “Play Kindly” from the perspectives of parents and professionals. These research methods are described in the “Design” section.

## Methods

### Intervention

**Figure 1:**
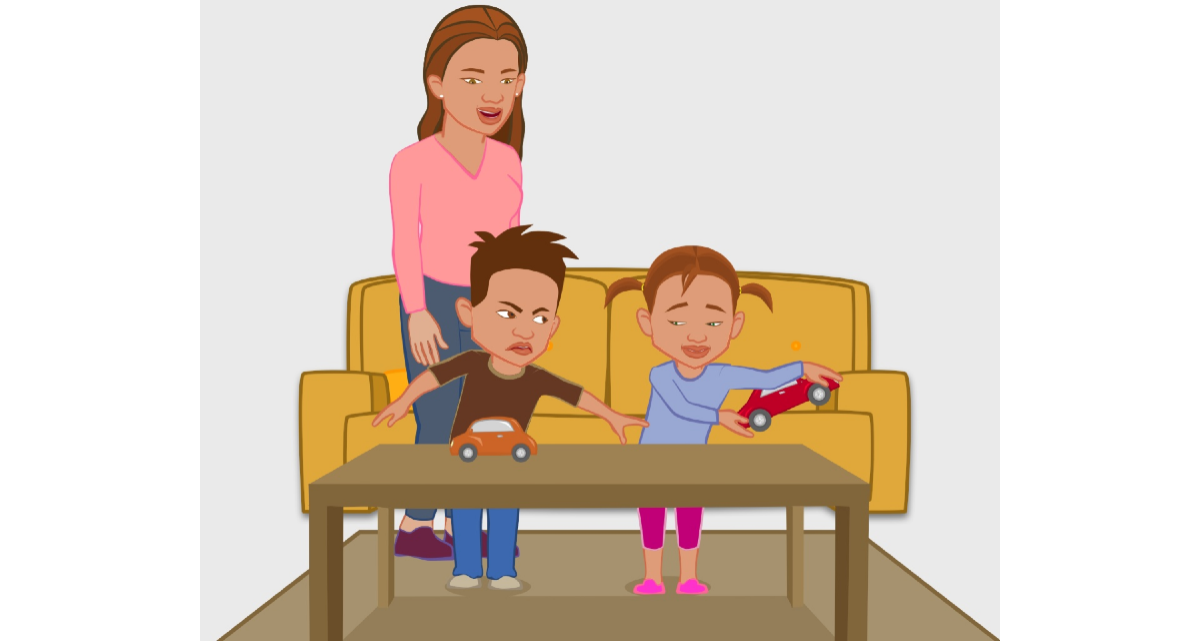
Frame from one of the parenting scenario animations in Play Kindly.

**Figure 2:**
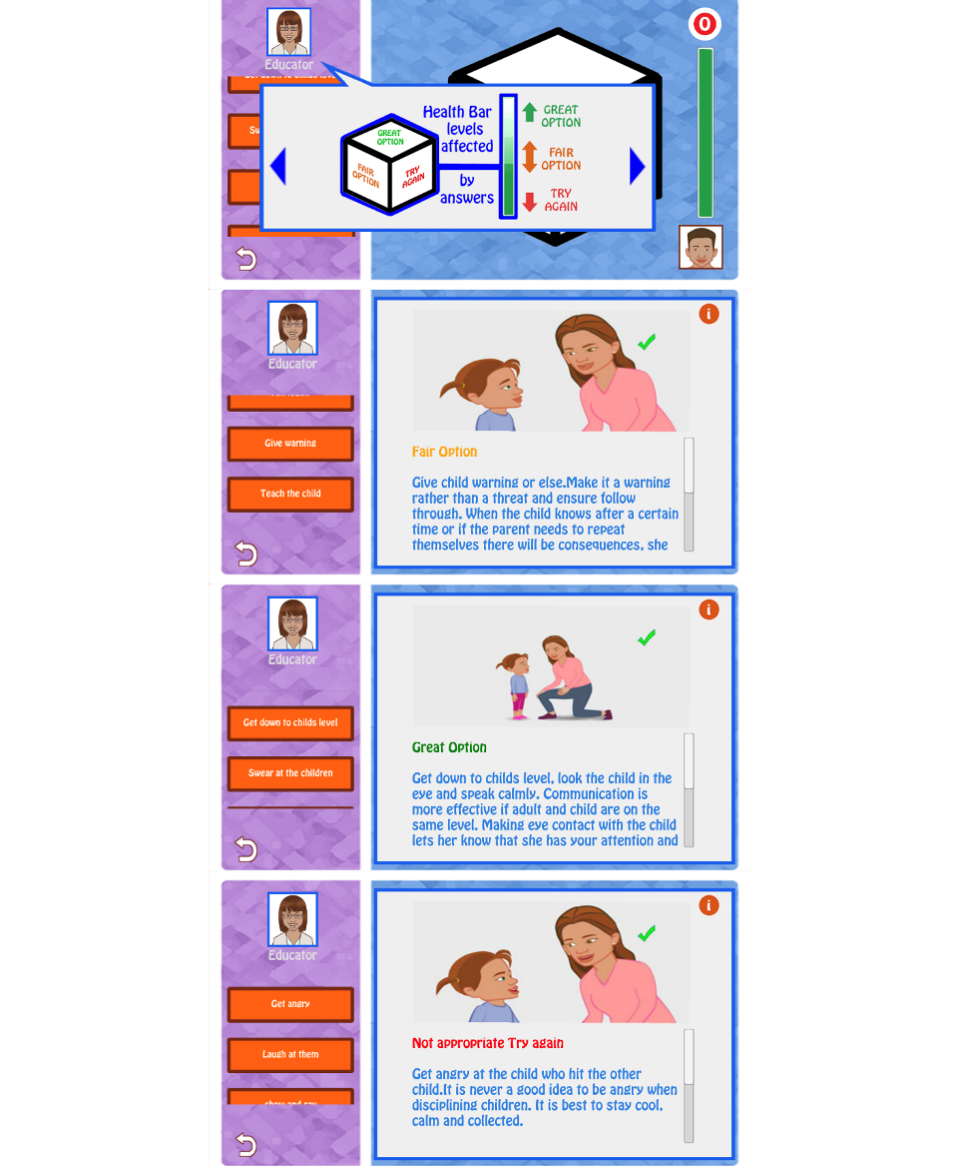
Play sequence from one of the Play Kindly scenarios.

Play Kindly is an interactive, cross-platform computer game designed for use on a smartphone. The interface is designed to resonate with Pacific people (Figure 1). The ideas, scenario scripts, narration, and artwork were undertaken by a team consisting of an established Pacific academic, a Pacific playwright, and a Pacific animator working alongside researchers and health professionals. Each scenario is short and lasts a few minutes. Parents can choose to play one scenario at separate times or all of them in one sitting. For each scenario, the participant chooses from a drop-down menu the most appropriate way to manage the behavior. Three “great” (correct) answers are needed to progress to the next scenario. Feedback about “fair” or “try again” answers are delivered by an “educator” avatar, who also gives instructions about how to play the game (Figure 2). Behaviors addressed in the game are *fights over belongings, refusing to eat, refusing to be dressed, and temper tantrums.* The strategies taught are predominantly focused on effective communication with the child, such as using a calm directive voice and eye contact, using distraction, and offering alternative strategies while not engaging in aggressive or harmful strategies.

The strategies in Play Kindly build on research with Samoan parents using the interactive CD-ROM tool “Play Nicely” [19]. The authors of the original “Play Nicely” described using content from parenting resources distributed by the American Academy of Pediatrics and American Psychological Association from around the time it was developed in the late 1990s, alongside input from multiple experts in the areas of psychology, pediatrics, and early childhood education [20].

The gamification and behavioral change techniques employed were the use of rewards such as badges, different levels of play, the use of avatars, the ability to customize some features, and feedback on performance.

### Design

The qualitative method was chosen to get an in-depth understanding about how the app was viewed by parents, particularly those groups underrepresented in health research, and professionals. Pacific parents for whom the app had been designed were the main target group. Our methodologies were, therefore, chosen to engage and work effectively and appropriately with Pacific and Māori communities, include both Pacific [21] and Māori [22] approaches, and take into account Pacific and Māori health research guidelines and qualitative research methods.

Pacific health research approaches are framed around the Pacific cultural values of communal relationships, reciprocity, holism, and respect from the beginning to the end of the research process [21]. The Pacific team, in partnership with a team from the University of Auckland, collaborated to cocreate an evaluation process that incorporated Pacific values and a rigorous scientific method. The cocreation process prioritized the concept of Teu le Vā, which involves identifying and understanding the “vā,” or spaces, between different stakeholders in Pacific research and development, and sharing knowledge across these spaces.

To establish the “vā,” engagement was in a face-to face manner with Pacific and Māori researchers, and incorporated a welcome ceremony, sharing of food, introductions, and a meaalofa (gift). This is also consistent with Māori tikanga protocols.

The Māori ethical framework shares many principles with Pacific guidelines and emphasizes whakapapa (relationships), tika (research design), manaakitanga (cultural and social responsibility), and mana (justice and equity) [22].

Collaboration across teams and communities, and adherence to cultural guidelines demonstrated our attempt to optimize relationships, enhance Pacific and Māori capacity and capability, and conduct research within a cultural framework.

### Participants

The five focus groups interviewed consisted of 2 contemporary pan-Pacific groups, 1 Māori group, 1 young Pacific adults or prospective parents group, and 1 open group. Pan-Pacific refers to individuals from the major Pacific ethnic groups in New Zealand. Contemporary refers to Pacific people predominantly fluent in English, who are more likely to have been born in New Zealand and belong to multiple ethnic groups. This subset were chosen because the majority (60%) of the Pacific population in New Zealand are in this category [15]. The open group refers to non-Māori and non-Pacific New Zealanders. Table 1 provides further details about each group.

**Table 1:** Type of group, numbers, sex, and ethnicity of participant focus groups.

Focus group	Participants, n	Female, n (%)	Male, n (%)	Ethnicity
Pacific focus group 1 (Pacific 1)	10	8 (80)	2 (20)	Tongan, Samoan, Samoan/European, Niuean
Pacific focus group 2 (Pacific 2)	11	8 (73)	3 (27)	Samoan, Tongan, Cook Island
Māori/Ngā Matua focus group (Māori)	6	5 (83)	1 (17)	Māori
Open focus group (open)	9	8 (89)	1 (11)	New Zealand European, South African
Pacific youth focus group (youth, 18-23 years of age)	9	4 (44)	5 (56)	Samoan, Tongan, Niuean, Cook Island

The 12 professionals interviewed individually were from a range of disciplines and had knowledge of child health and development, parenting interventions, or Pacific health. Some professionals had extensive experience in implementing evidence-based parenting interventions. The largest number of respondents were Samoan or New Zealand European. Table 2 provides further details of the professionals who conducted the interviews.

**Table 2:** Profession, ethnicity, and number of interviewees in the professional interviews.

Professional background	Participants interviewed, n	Ethnicity
Psychologist	5	3 NZE^a^, 1 Samoan, 1 Māori
Pediatric doctor	2	1 Māori, 1 European
Research and teaching	2	2 Samoan
Early childhood education	1	1 NZE
Nurse with qualifications in child health	1	1 Asian
General practitioner	1	1 Fijian

^a^NZE: New Zealand European.

### Recruitment

Pacific research team members recruited parents through snowball sampling. A total of 3 people known to one of the research assistances were contacted by telephone. The research assistant gave them information about the study, asked whether they would like to take part, and asked them to identify other potential participants. These people were contacted in the same way. This process continued until the researcher had all the participants needed.

Snowball sampling is a technique commonly used when a sample of the study is limited to a small subgroup of the population and starts through known acquaintances that fit criteria. The researchers typically ask for assistance from the participants recruited so far to help identify other people with a similar trait or interest. This method was chosen because of historical difficulties in recruiting Pacific and Māori people to take part in research. These communities have reported feeling overresearched and underinvolved in the design and feedback from research in general. This method meant that on two occasions the interviewee knew one of the research assistants, an almost unavoidable circumstance given the small and often well-connected Pacific community, so this research assistant did not conduct the interviews. A total of 55 individuals were approached; 3 individuals did not respond to the invitation to take part, and 7 did not attend the focus group interview as arranged.

Professionals were recruited by expert sampling**,** a form of purposive sampling needed to capture the specific expertise required to answer questions about content and cultural appeal. Expert status was defined as a person, preferably of Pacific decent with extensive knowledge based on research or occupation in the area of child health. Names of individuals were put forward by academic staff familiar with those working in the field across New Zealand. These individuals were contacted by phone or email and asked if they would like to take part. The number of professionals interviewed was determined by the emergence of data saturation. One professional knew one of the app designers and was reminded that their responses would be anonymized and kept confidential. By the end of the study, 20 professionals had been contacted by email; 7 were too busy to take part, and 1 stopped responding to emails.

### Procedure

All focus groups were conducted in English. The focus groups were run by 1-2 Pacific researchers or 1 Pacific researcher and 1 European or New Zealand European researcher. The researchers held either an MD or PhD, or worked in the field of child mental health (psychiatrist or psychologist). Similarly, the Māori focus group was cofacilitated by a Māori facilitator to ensure that Kaupapa Māori research processes were followed. All interviewers were employed as researchers by the University of Auckland with the exception of the Māori facilitator. None had financial interests in the success of the app. The Pacific facilitators knew the app designers and had an interest in Pacific health initiatives in New Zealand.

Semistructured interviews were chosen to ensure that the aims of the study were addressed but gave space for new themes and ideas to emerge. In line with Kaupapa Māori and Pacific research methodologies and frameworks, focus groups included a welcome ceremony, the sharing of food, introductions, and setting the scene for the research.

Prior to the focus group or interview, all participants were asked to complete the app. If parents had not looked at the app before the interviews, the research team allowed time for them to start playing it on provided tablets before they commenced, not necessarily finishing the whole app. The focus groups lasted between 45 minutes and 1 hour, and were all face-to-face. The professional interviews lasted between 30-40 minutes and were conducted at the person’s home or workplace, or over the phone. All professionals had familiarized themselves with the app by the time of the interview. Not everyone had completed all of the scenarios due to usability issues. The interviews were digitally recorded and transcribed verbatim. Script and audio were reviewed by Pacific and Māori researchers to ensure accuracy in interpretation. Transcripts were returned for participants to comment.

### Data Analysis

All focus groups and individual interviews were recorded, and a general inductive approach was used for data analysis. This approach included collecting and becoming familiar with raw data, generating and revising initial codes, searching for themes, and then reviewing and defining themes and subthemes. Codes and themes were derived from the raw data and coded using NVivo software (QSR International) [23].

The coding was carried out by 1 Pacific, 1 European, and 1 Māori researcher. An initial meeting took place to discuss ideas about codes relevant to the research question. Following this, each researcher worked on transcripts separately coding everything relevant to the research question. The Māori researcher only coded the Māori focus group manuscript alongside the Pacific and European researcher. This was to ensure that codes and themes were interpreted correctly, taking into account the cultural context. Overall, 40% of the data was independently coded by at least 2 researchers, and any disagreements were resolved by discussion. The codes were then organized into broader themes related to the research question. These were reviewed and modified until it was felt that the data accurately supported the data and captured the entire data set.

### Ethical Approval

Ethics approval for this research was obtained through The University of Auckland Human Participants Ethics Committee (Ref 018135).

## Results

Results are summarized in Table 3 across themes that were identified of acceptability, usability, cultural specificity, and content.

The Pacific theme and real-life nature of scenarios appealed to most regardless of ethnicity. There was some disagreement about how culture specific the app should be, and opinions were likely to be influenced by how strongly the person identified with their own culture. The involvement of an artist and playwright helped to create something that felt real, but the light-hearted approach began to get lost as professionals tried to deliver parenting advice. The main point of contention for some was the virtual coach (avatar) who was referred to as an “educator.”

The main concerns about content were parenting advice not being tailored for individual families, not taking into account the context in which the behavior occurred, and not providing sufficient instruction to teach strategies effectively. Addressing this could be challenging, as longer explanations would detract from the game, and video demonstrations would use too much data.

**Table 3:** Major themes, subthemes, descriptions, and examples from the data.

Theme and subtheme		Examples
**Acceptability**		
	Visual appearance (mixed opinion)	“I like the visuals, kind of simple, it’s not too complex looking” (Pf^a^3) “It looks like it’s developed in the 90’s, a bit old” (Māori)
	Relating to scenarios	“It didn’t shy away from the reality, particularly the responses of Pacific families” (Pf3) “It has everyday things that parents come up against” (Open)
	Delivery of parenting education	“There was kind of like academics trying to get down with the blue collars workers and you go, that would never happen in real life” (Māori) “Why is this person (avatar) called an educator? It feels you are doing something wrong” (Youth) “I thought it was quite informative, I wouldn’t even think to do that” (Open)
**Usability**		
	Repetitive	“It’s like ok I got it the first time...every time it keeps coming up...and I keep scrolling to the end...and I have to do it again...yea I got it the first time” (Pacific 2)
	Flow	“Maybe the flow wasn’t so smooth, like it seemed a bit jittery and maybe fragmented, like no real flow going into each phase” (Pacific 1)
	Progressing to the next level (unclear instructions)	“Like I couldn’t get past it, like I did the first scenario and I thought the little explanation like why those decisions are the best I really thought that was informative but I kind of couldn’t get past this part” (Māori)
**Cultural specificity**		
	Varying attitudes	“There was a link to a Pacific peoples/Kiwis that you could actually connect with” (Pf1) “Yes, there were brown faces, but that was about it. The rest was very westernized and clinical” (Pacific 1) “Māori people would want Māori speaking, Māori looking things. Other Māori, bicultural Māori from bicultural and things would be ok with, they’ll just take what they need ‘cos they feel kind of safe and walk in both worlds” (Māori) “Parenting isn’t cultural, parenting is parenting” (Pacific 2)
**Content**		
	Incremental challenges (unable to progress to the next level without 3 “great” answers)	“There’s a bit of an impression from it that there is a right way and a wrong way, and I personally don’t feel particularly comfortable with that” (Pf2) “I put ignore the behaviour which is what I have been doing with my kids and they stop and it went orange (fair answer) and then I went oh?” (Māori)
	Amount of text	“Like one (of the options) was ‘voice’ I was like, oh, okay I use my voice?” (Pacific 1) “Get down to the child’s level’, people might think, well, what do you mean, do I talk like my four-year-old? Do I do what they’re doing?” (Pf5) “It would be great to have a video of someone going, this is why you get down to their level because of this, you know what I mean, because people don’t like to read, I don’t like to read” (Pacific 1)
	Overly simplistic	“It doesn’t privilege your voice as a parent already” (Open) “The focus is more about talking with the family about what’s working for them, what are the solutions they’ve tried...rather than going there is a certain way to deal with this particular situation, so it wasn’t a good fit for me” (Pf7)
	Important information missing	“I felt like there was some fundamental information that was needed first of all...even at like a really basic behavioural level, what you pay attention to is what you’ll get more of” (Pf4) “So definitely the front and centre stuff around building relationships, that’s the big thing for me that I think’s absent from this” (Pf5)

^a^Pf: professional.

To progress to the next scenario, parents needed to choose three “great” answers, but this was often not clear to them. Professionals felt that distinguishing between a “great” and a “fair” answer was unhelpful because perfect parenting was idealistic. There was also some disagreement about what should constitute a good or fair answer. Professionals were concerned about the effect on struggling parents who could not progress through the scenarios and how this might impact their view of themselves as parents.

Finally, professionals recommended that the app should focus on an aspect of parenting rather than general parenting. They suggested the inclusion of what they regarded as the most important strategies: playing with your child and positive reinforcement.

## Discussion

### General Discussion

This is the first time a parenting app has been designed for Pacific parents and the first time feedback from this important user group and from Māori parents has been sought. Parenting is a sensitive subject and this study highlighted the level of thought needed to deliver advice in a palatable way. Perhaps those searching for a parenting app to upskill would be less likely to object to this method of information delivery than those that participated in the groups who may not have ordinarily sought out such an app. Collaborating with marketers, game designers, and parents in the design process could help to set the correct tone.

The techniques to “gamify” the app, such as incremental challenges, a scrolling bar of options, and the use of a virtual coach, had the potential to engage with or discourage parents. Our findings support previous research that gamification techniques are only successful if gaming principles like motivational affordances (eg, levels, achievements, feedback) are implemented with specific behavioral outcomes in mind [24,25]. This further highlights that scientific institutions need to work with art industries, game developers, and marketers to create something that is acceptable to the general public and leads to behavioral change.

Concerns about the app not taking into account the context in which the child’s behavior occurred and not sufficiently explaining the strategies could be mitigated by the fact that the app is aimed at children who have everyday behavioral problems. The level of intervention needed would, therefore, be low. Targeting this cohort, even if the information is brief, could prevent more significant problems from developing or act as a stepping stone for further support. The professionals’ suggestions of narrowing the focus to two important strategies could potentially widen the reach of the app to parents of children with behavioral problems of different severity and could also address the issues with the amount of instructions and text.

The feedback from the professionals and parents was useful in providing a clear framework for adaptation. Play Kindly was developed on a modest budget, and many of the usability difficulties experienced could be addressed as further funding was secured. There is an increasing recognition of the importance of co-design, and greater use of this with parents and professionals at the start might also have mitigated some of the perceived problems.

### Limitations

This was a small study with a sample size that is consistent with design research [26], so findings are not intended to be generalized to the population of parents in New Zealand. The diversity of the sample was compromised by snowball sampling. Two people were known to the one of the researchers, so it was ensured that the person who conducted the interview was unfamiliar to them. One participant knew one of the app designers and admitted feeling anxious about expressing their views. This was addressed by reminding them that their opinions would be confidential and anonymized. Without the involvement of a Pacific research team, it is unlikely that a sufficient number of participants would have been recruited to this study, and thus, bias was difficult to avoid. The researchers who conducted the interviews formed their own opinions about the app on the basis of the reviews from parents and professionals. This bias was reduced by asking for other members of the research team, not present at the interviews, to read the transcripts and compare with the overall report.

Many parents and professionals did not play the entire game because of usability issues and not picking the correct answers as deemed by the app. It is, therefore, possible that a number of themes were missed. The issues raised here will be addressed once the app has been modified and retested by reinterviewing users of the app.

### Future Directions

This study lays the groundwork for further redevelopment of the app with the feedback obtained through the qualitative paper described here. After redevelopment, the revised app will be trialed in a feasibility trial with further refinement following and then a controlled larger scale trial. Involving stakeholders in the design of electronic interventions is highly recommended for future projects of this nature.

### Conclusions

Play Kindly is the first culturally adapted parenting app of its kind designed to target Pacific parents and other New Zealanders. Aimed at everyday parenting problems, this app has potential in Pacific communities where there is limited culturally specific parenting resources and could also appeal to other New Zealanders through its visual appeal and relatability.

Iterative design is a core part of the development of digital technologies. Play Kindly was appealing, and with further development to increase the content as recommended by the professionals and address some of the usability difficulties, it appears to have the potential to be a useful tool for parents of young children.
